# Transcriptome-Guided Drug Repositioning

**DOI:** 10.3390/pharmaceutics11120677

**Published:** 2019-12-12

**Authors:** Arsen Arakelyan, Lilit Nersisyan, Maria Nikoghosyan, Siras Hakobyan, Arman Simonyan, Lydia Hopp, Henry Loeffler-Wirth, Hans Binder

**Affiliations:** 1 Institute of Biomedicine and Pharmacy, Russian-Armenian University, 0051 Yerevan, Armenia; marianikoghosyan@gmail.com (M.N.); rmnsimonyan@gmail.com (A.S.); 2 Group of Bioinformatics, Institute of Molecular Biology NAS RA, 0014 Yerevan, Armenia; l_nersisyan@mb.sci.am (L.N.); sirashakobyan@gmail.com (S.H.); 3 Interdisciplinary Centre for Bioinformatics, University of Leipzig, D-04107 Leipzig, Germany; hopp@izbi.uni-leipzig.de (L.H.); wirth@izbi.uni-leipzig.de (H.L.-W.)

**Keywords:** drug repositioning, transcriptome, self-organizing maps, ulcerative colitis, Crohn’s disease, COPD, sarcoidosis, psoriasis, systemic juvenile idiopathic arthritis

## Abstract

Drug repositioning can save considerable time and resources and significantly speed up the drug development process. The increasing availability of drug action and disease-associated transcriptome data makes it an attractive source for repositioning studies. Here, we have developed a transcriptome-guided approach for drug/biologics repositioning based on multi-layer self-organizing maps (ml-SOM). It allows for analyzing multiple transcriptome datasets by segmenting them into layers of drug action- and disease-associated transcriptome data. A comparison of expression changes in clusters of functionally related genes across the layers identifies “drug target” spots in disease layers and evaluates the repositioning possibility of a drug. The repositioning potential for two approved biologics drugs (infliximab and brodalumab) confirmed the drugs’ action for approved diseases (ulcerative colitis and Crohn’s disease for infliximab and psoriasis for brodalumab). We showed the potential efficacy of infliximab for the treatment of sarcoidosis, but not chronic obstructive pulmonary disease (COPD). Brodalumab failed to affect dysregulated functional gene clusters in Crohn’s disease (CD) and systemic juvenile idiopathic arthritis (SJIA), clearly indicating that it may not be effective in the treatment of these diseases. In conclusion, ml-SOM offers a novel approach for transcriptome-guided drug repositioning that could be particularly useful for biologics drugs.

## 1. Introduction

Drug repositioning (repurposing) is the identification of alternative target diseases for already approved drugs, which can save tremendous resources and significantly speed up the drug development process [1,2]. Currently, computational drug repositioning relies on several main strategies or their blends (reviewed in [2]). The first strategy employs structural bioinformatics approaches (molecular modeling, dynamics simulations, and docking) to identify compound–target pairs [3]. Though it has demonstrated its efficacy in several studies, the main limitation of this approach is the knowledge of the chemical structure of the drug and the 3D structure of its target. This, in turn, severely impedes the repositioning potential of structural bioinformatics approaches. Particularly, for the category of drugs known as biologics, such as vaccines, blood components, allergenics, somatic cells, gene therapy, tissues, and recombinant therapeutic proteins and antibodies, their structural models are not available, thus making structural bioinformatics-based repositioning studies not suitable [4,5].

Transcriptome based approaches employ analysis of gene expression and identification of molecular targets that show anti-correlated differential expression in disease and upon drug administration [6]. These methods are not aimed at the identification of molecular targets through drug binding sites, rather they focus on identification of diseases, which share molecular pathomechanisms that are targeted by the given drug. Pathway and gene set enrichment-based repositioning approaches can be considered as an extension of transcriptomic methods since the first step in those methods is identification of differentially expressed genes (DEGs) that is followed by functional annotation of biological processes and pathways [7,8]. The overlapping processes and pathways are used for assessment of disease–disease, gene–disease, and drug–disease associations.

Transcriptome and gene set based repositioning pipelines are mainly performed by matching a list of deregulated genes against genes in drug-perturbation profiles, as in the connectivity Map project [9] and NFFfinder [10]. Other methods employ correlation [11], network [12], and graph-based analyses [13] and others reviewed in [14,15]. The main limitation of these methods is that only overlapping sets of genes or processes that describe the main drug target mechanism are scored for assessment of repositioning “potential” for a drug, while the off-target and alternative processes and/or pathways involved in disease pathogenesis or affected by the drug are mainly left aside. The latter may overtake drug-actions or cause adverse effects that are important to consider. This necessitates the development of methods that allow for a holistic view of full transcriptome landscapes and for disentangling functional modules of dysregulated genes in diseases and upon drug action.

Here we propose a transcriptome based approach for drug repositioning, which is based on a multi-layer self-organizing maps (ml-SOM) algorithm for clustering transcription profiles of genes in healthy and disease conditions and upon drug action. ml-SOM is an extension of a previously developed SOM based transcriptome analysis pipeline [16,17] that has been used for comprehensive analysis of transcription landscapes in cancers and other chronic diseases and proven to be valuable for the general understanding of disease pathomechanisms, identification of molecular subtypes, biomarker selection, and functional information mining [18,19,20,21].

## 2. Materials and Methods

### 2.1. Prediction for Drug Repositioning Based on Expression Signatures

Our prediction method assumes that a certain disease (D1) induces alterations of gene regulatory programs resulting in differential expression (DE) of a number of disease-associated functional gene modules with respect to the healthy reference state. An effective drug and/or treatment (T1) then is expected to reduce or even to completely compensate for this disease-related DE. We further assume that if another disease (D2) shares the same or similar pathomechanisms with D1, then it eventually will affect gene regulation in a similar way and, consequently, the expression of virtually the same set of signature genes will alter in D2 compared with the respective reference state. Based on the assumption of equal pathomechanisms, drugs/treatment targeting the D1 (T1) are also expected to target D2, i.e., we assume T2 = T1. In other words, T1 then constitutes a candidate for drug repositioning for treating D2. Hence, drug repositioning based on expression signatures requires (i) identification of a set of signature genes specifically affected by D1; (ii) effect-compensation of these signature genes by T1 and, (iii) identification of the same pathomechanisms in D2 as in D1 by DE analysis based on signature genes overlapping between D1 and D2.

### 2.2. Experiment Design and Datasets

**Figure 1:**
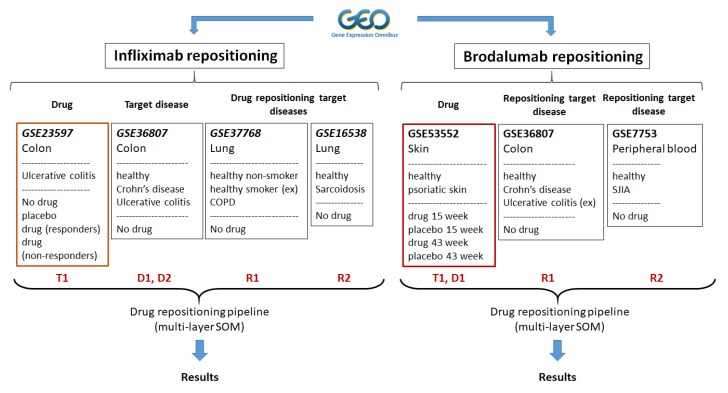
The experimental design. Two independent studies were performed. In the first study, we combined datasets to study the potential of infliximab repositioning in chronic obstructive pulmonary disease (COPD) and pulmonary sarcoidosis; in the second study, we combined datasets to study brodalumab repositioning in Crohn’s disease and systemic juvenile idiopathic arthritis. T1—treatment data set, D1 and D2—target disease datasets for T1, R1, and R2—repositioning disease data sets. Ex—excluded from the analyses.

Our method for drug repositioning thus requires at least three types of case-control “basic” datasets (that could also be partially combined):

- T1 (treatment data set, and potentially additional data sets T2, T3, etc.)—baseline (untreated disease samples) vs. treated samples;
- D1 (target disease dataset, and potentially additional target diseases D2, D3, etc.)—disease (untreated samples) vs. control (healthy) samples;
- R1 (repositioning disease dataset, and potentially additional diseases R2, R3, etc.)—disease (untreated samples) vs. control (healthy) samples;

In our study, we have used transcriptome datasets taken from the Gene Expression Omnibus (GEO) [22] to evaluate repositioning after treatment with two biologics drugs (antibodies), namely infliximab and brodalumab (Figure 1).

In Study 1 we used disease and treatment data to evaluate the effect of infliximab (T1) on gene expression signatures of ulcerative colitis (D1) and Crohn’s disease (D2) and the possibility of repositioning as potential therapeutics in chronic obstructive pulmonary disease (R1) and pulmonary sarcoidosis (R2). Since infliximab is an anti-TNFalpha antibody, we chose chronic obstructive pulmonary disease (COPD) and sarcoidosis as repositioning targets since this cytokine is implicated in their pathology [23,24,25,26].

The following datasets were included in this experiment:

- GSE23597: Expression data from colonic biopsy samples of infliximab treated UC patients. The dataset contained expression profiles of colonic biopsy samples from 48 patients with moderate-to-severe ulcerative colitis (UC). Patients were treated with infliximab (5 or 10 mg/kg) or placebo at weeks 0, 2, 6, and every 8 weeks thereafter (total duration 46 weeks). One hundred and thirteen biopsy samples were collected at baseline (before starting treatment), and at 8 and 30 weeks. Infliximab treated patients were further stratified into responder and non-responder groups [27].
- GSE36807: Genome-wide analysis of Crohn’s disease and ulcerative colitis biopsy samples. The dataset consisted of gene expression patterns of 13 Crohn’s disease (CD), 15 ulcerative colitis (UC), and 7 healthy control colonic specimens [28].
- GSE37768: Expression data from lung tissue of moderate chronic obstructive pulmonary disease (COPD) patients, healthy smokers, and non-smokers. The dataset consisted of global transcriptome profiling of peripheral lung tissue samples from moderate COPD patients (*n* = 13), healthy smokers (*n* = 11), and nonsmokers (*n* = 9). The healthy smoker group was not included in this study since no information about smoking status was available for COPD patients.
- GSE16538: Genome-wide gene expression profile analysis in pulmonary sarcoidosis. The dataset consisted of gene expression profiles of lung tissues from patients with pulmonary sarcoidosis (*n* = 6) and healthy controls (*n* = 6) [29].

In Study 2, we used disease and treatment data to evaluate the effect of brodalumab (T1) on gene expression signatures of psoriasis (D1) and the possibility for repositioning as potential therapeutics in Crohn’s disease (R1) and systemic juvenile idiopathic arthritis (SJIA) (R2). Brodalumab (anti-IL-17 receptor A antibody) was approved for the treatment of severe-to-moderate psoriasis and psoriatic arthritis [30]. We chose Crohn’s disease (CD) and systemic juvenile idiopathic arthritis (SJIA) as repositioning targets since IL-17 signaling is implicated in these diseases [31,32].

The following datasets were included in this experiment:

- GSE53552: Gene expression profiling from psoriatic lesional and non-lesional skin [brodalumab treatment]. The dataset consisted of 99 skin biopsy samples from 25 patients obtained at treatment baseline and on days 15 and 43 (total duration 6 weeks). Patients were treated with brodalumab in doses of 140 mg s.c., 350 s.c., and 700 mg i.v. and placebo [33].
- GSE36807: Genome-wide analysis of Crohn’s disease and ulcerative colitis biopsy samples. The dataset description is provided in Experiment 1. Only Crohn’s disease and healthy control colonic samples were used.
- GSE7753: Gene expression profiling in peripheral blood in untreated new-onset systemic juvenile idiopathic arthritis (SJIA). The dataset consisted of gene expression profiles from peripheral blood mononuclear cells of 17 new-onset SJIA patients and 30 normal controls [34].

All the datasets were generated using the Affymetrix Human Genome U133 Plus 2.0 arrays (GEO accession GPL570). Raw Affymetrix CEL files were downloaded for all the datasets. Probe signal intensity conversions, RMA normalization, and chip annotation were performed using the “affy” package for R [35].

### 2.3. Validation of Drug Target Pathway Signatures

To confirm the drug action, we evaluated the changes in drug-associated gene expression signatures in target and repositioning disease data sets in a first step before proceeding to the repositioning analyses. Since both drugs under consideration are biologics and target specific pathways, namely TNF-alpha signaling for infliximab and IL-17 pathway for brodalumab, we validated their functional state by performing gene set analysis using Z (GSZ)-score metrics of gene sets characterizing the respective pathways [36,37]. For GSZ analysis of TNF-alpha- and IL-17-related gene sets we used precollected gene lists included in the oposSOM package that was populated from various sources, including the Gene Ontology [38], GSEA-repository [39], the Human Protein Atlas: Tissue Atlas [40], as well as selected scientific publications [18,36,41].

Topology based evaluation of pathway activity was performed using the Pathway Signal Flow (PSF) algorithm developed previously [42] and further implemented in the oposSOM package [43]. PSF takes as input gene expression fold change values of pathway nodes and evaluates the flow of signal in the direction from pathway inputs to outputs via pairwise interactions between the nodes depicted in the pathway topology. The interactions of an activating nature multiply the signal, while the inhibitory ones reduce it, reaching a PSF value indicating overall pathway activity in the final sink nodes [42].

### 2.4. Multi-Layer Self Organizing Maps (ml-SOM)

Our drug repositioning approach requires robust identification of expression signatures and of their activation in the different data sets referring to the reference disease (D1), its treatment (T1), and diseases presumed for repositioning (R1). We made use of self-organizing maps (SOM) portrayal method, which has been proven to robustly identify sets of co-expressed genes in large scale transcriptome data [16,18,36,41]. SOM portrayal treats genes as objects and their expression profiles across samples as features. SOM clusters objects with similar features (i.e., co-expressed genes) together into microclusters called “metagenes”, arranges them on a two-dimensional grid, and colors metagene expression in blue to red scale for low-to-high expression values, respectively. Due to self-organizing properties of SOM, metagenes of similar profiles aggregate together into spot-like areas representing clusters of co-regulated metagenes with associated single genes [16,36]. Spots typically gather metagenes centered around one or several biological functions according to the guilt-by-association principle [44].

**Figure 2:**
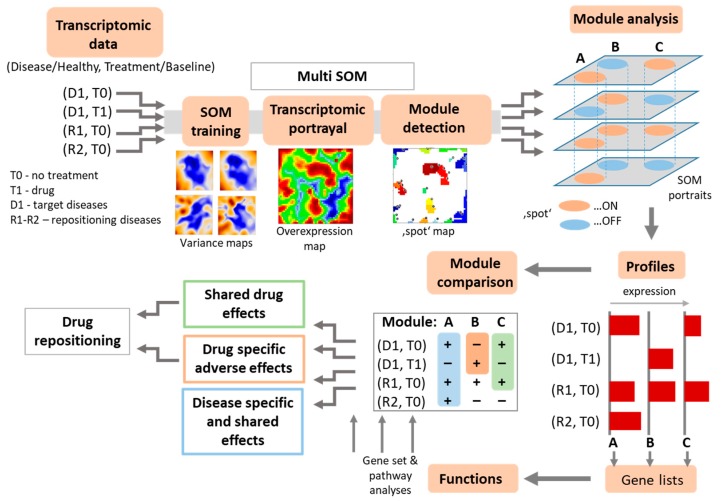
The workflow of multi-layer self-organizing maps (ml-SOM) based drug repositioning. The layers of disease and drug action transcriptome datasets were normalized, scaled and concatenated, and afterwards subjected to SOM training. The SOM algorithm produced sample-wise transcriptomic portraits by combining genes with similar expression profiles across all the layers and by using blue (underexpression) to red (overexpression) color scale to visualize them. A global expression summary map was then produced from individual portraits, and spot-like modules of dysregulated genes were detected. These modules were then evaluated for identification of disease- or drug-response associated gene clusters and for evaluation of drug repositioning potential.

For drug repositioning purposes, we modified the SOM method in such a way that each of the transcriptome datasets referring to different diseases and treatments were arranged into one separate layer of SOM-processed data in a multilayered assembly (ml-SOM). An essential requirement for the datasets used with ml-SOM is the presence of samples from one or several target conditions (i.e., disease, or drug), as well as of reference samples (i.e., healthy controls for diseased and baseline and (or) placebo samples for drug action) on each layer. The workflow of the ml-SOM approach is presented in Figure 2. In a first step, transcriptomic data were preprocessed, which includes global centralization of each gene over all datasets, independent quantile normalization of each dataset, and their mutual harmonization to prevent the overweighting of any layer in the training process, as described previously [45]. Next, all the datasets were trained together into a unique SOM of size 45 × 45 metagenes using default SOM parameters settings as implemented in the oposSOM package [17]. Each of the layers was characterized by a series of supporting maps, such as population, variance, and overexpression maps, as described previously for single-layer SOM [17]. As a result of this combined SOM training, each of the layers was projected into identical SOM-space formed of metagenes that contain the same single genes at the same position of the metagene-grid in each of the layers which made them directly comparable across the layers.

### 2.5. Spot Calling

**Figure 3:**
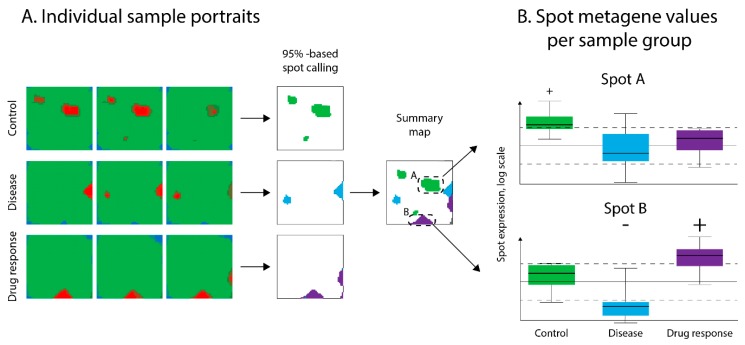
Detecting spot-modules of co-regulated genes in SOM portraits. (**A**) Spot detection on sample SOM “portraits” from all layers was performed using “overexpression” criteria defined as clusters of metagenes with values higher than the 95th percentile of metagene value distribution. These spots were then transferred to the global summary map to provide an overview of the entire transcriptomic landscape. (**B**) Evaluation of spot over- or under-expression in samples was performed by comparison of the mean value of metagenes in a spot with defined thresholds (see Methods section). Spots A and B were arbitrarily selected for demonstration purposes.

After SOM training, the resulting metagene map was segmented into so-called “spots”, representing clusters of co-expressed genes overexpressed in at least one of the data sets. Spots were detected in the individual SOM-portraits using an “overexpression” criterion. Particularly metagenes with expression values exceeding the 95th percentile of the overall distribution of metagene values were considered as overexpression spots [16]. The spots were then transferred into an overexpression summary map (Figure 3A), which provided an overview of the transcriptomic landscape of all the layers [16]. The expression values of each spot detected across the different layers constituted the respective spot profile (Figure 3B). Genes associated with spots were subjected to functional annotation with Fisher exact test-based gene set enrichment analysis using the oposSOM package [17].

### 2.6. Differential Spot Expression Analysis

**Figure 4:**
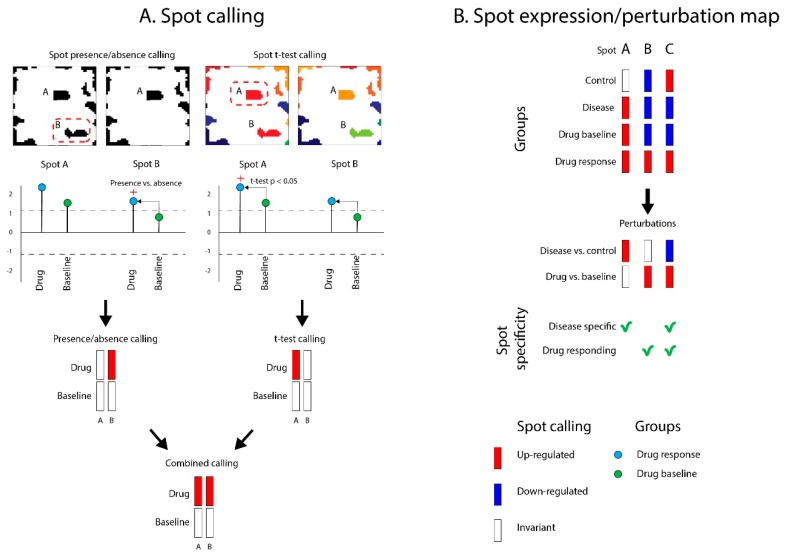
Spot calling for drug repositioning and prediction. For demonstration purposes, we present here a hypothetical case of spot calling on drug data layers. (**A**) Dysregulated spots are “called” across the layers by differential analysis using presence/absence(with ± SD of the spot metagenes expression in the given layer as criterion) and *t*-test calling approaches (using combined *p* < 0.05 and spot fold-change > 1.5 criteria) that evaluate the difference between target (drug action in this example) and reference states (drug baseline in this example). The votes are finally combined into a unique one. (**B**) Spot calling is performed on all data layers (drug and disease) and results in a spot perturbation heatmap, which is used for drug repositioning prediction.

In our study design, each layer contains samples grouped into the target (disease or drug) and reference states (healthy samples or drug baseline and (or) placebo). Spot detection on these layers resulted in a list of characteristic spots and their expression values for each studied sample group across all layers. To perform drug repositioning, we first identified spot modules that were up- or downregulated in the target (drug or disease) compared to reference groups (healthy controls or baseline/placebo) in each layer. Detection of such “differentially regulated” spots was performed using a combination of two approaches (Figure 4A). The so-called “presence/absence” calling of differential spots relies on the detection of a difference in the presence/absence of spots between target and reference states using a threshold of ±one standard deviation (SD) of spot metagenes expression in the corresponding layer [16,36]. The second “*t*-test” calling approach detected differences in spot mean expression values between target and reference states with Student’s *t*-test (using combined *p* < 0.05 and spot fold-change > 1.5 criteria) (Figure 4A). The results of both calling methods were then combined into a joint vote for spot’s presence/differential regulation in target groups for each layer. These assignments were then visualized as “spot perturbation” heatmaps (Figure 4B). Depending on the dataset layer, we assigned “disease spots” as those spots that changed in disease compared to healthy state, while we referred to “drug spots” as those spots that changed in drug compared to baseline.

Next, our drug repositioning strategy relied on the identification of those spots which were antagonistically regulated between disease and treatment groups. In case such spot(s) existed, the drug used for treatment was considered suitable for repositioning. The validity of drug–disease associations was evaluated based on published literature describing the effects of drugs on target diseases, as well as drug–disease labels available from the U.S. Food and Drug Administration (FDA) (https://labels.fda.gov/).

### 2.7. Data Availability

The complete analysis results were deposited as supplementary datasets in the open-access repository Zenodo (https://zenodo.org/) [46].

## 3. Results

### 3.1. Evaluation of Infliximab as a Potential Therapeutics for Ulcerative Colitis, Crohn’s Disease, COPD, and Sarcoidosis

To evaluate the effect of infliximab (T1) on UC (D1), CD (D2), COPD (R1), and sarcoidosis (R2) we performed ml-SOM training of four transcriptomic datasets, including one drug response and three diseases-related datasets (see Methods, subsection Experiment Design and Data Sets). Infliximab is a chimeric monoclonal antibody, which possesses anti-TNF-alpha activity [47]. The mechanisms of infliximab activity include binding to free and membrane-bound TNF-alpha, induction of apoptosis in T cells, inhibition of cytokine and chemokine expression, as well as cell–cell signaling and immune cell trafficking [27]. The Active Ulcerative Colitis (UC) Trial 1 (ACT1) study, as well as several other trials, have proven that infliximab is effective for the treatment of moderate to severe UC [48]. Crohn’s disease (CD) is also considered as a target for infliximab, and its efficacy was also demonstrated in clinical trials [49]. Since COPD and sarcoidosis are also characterized by an abnormal inflammatory response [20], particularly mediated by TNF-alpha and other cytokines [50,51], we aimed to evaluate similarities of transcriptome landscapes in UC, CD, COPD, and sarcoidosis and the ability of infliximab to target pathological processes in COPD and sarcoidosis as well.

**Figure 5:**
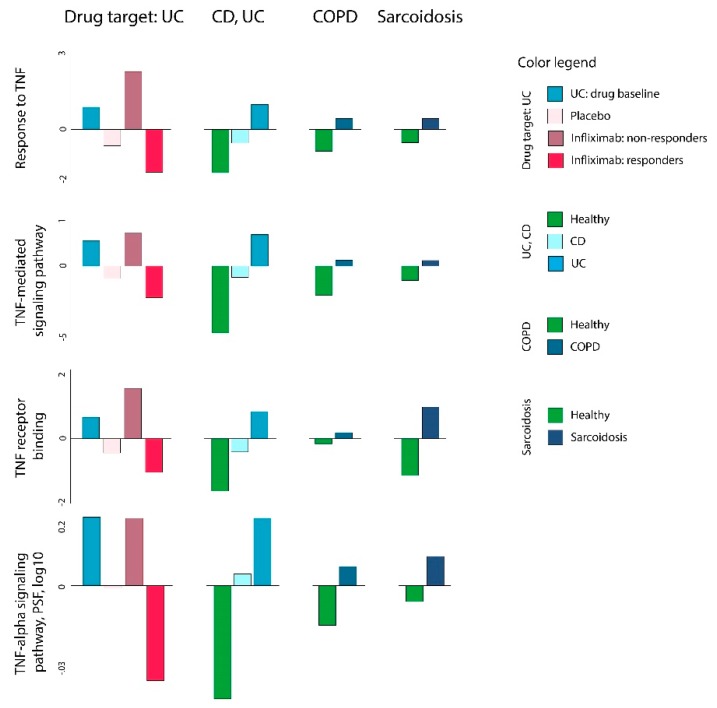
Gene set Z-score and pathway signal flow (PSF) analysis of TNF-alpha associated gene sets in ulcerative colitis (D1), Crohn’s disease (D2), COPD (R1), and sarcoidosis (R2) as well as infliximab (T1) datasets. TNF-alpha associated sets were chosen from the gene set collection available in the oposSOM package [17]. TNF becomes activated in all diseases compared to the respective healthy states. Treatment with infliximab virtually reverses this effect in responder UC patients. The common TNF activation in COPD and sarcoidosis makes these disease candidates for infliximab repositioning.

To prove this functional action, we first evaluated if the expression of TNF-alpha related gene sets and pathways [36,42] in the groups of the studied samples was potentially targeted by the drug before proceeding to the ml-SOM based repositioning pipeline. Indeed, using the GSZ-score and PSF approaches, we found that the TNF-alpha related gene signatures were upregulated in disease vs. healthy groups, and they were downregulated in response to infliximab (Figure 5, Supplementary Figure S1). Notably, the downregulation of these gene signatures was also observed in the placebo group, though to a lesser extent. The mechanism of this effect is unknown. However, recent studies have shown that in inflammatory bowel diseases, placebo effects, including remission and endoscopic response, are remarkable [52,53,54]. This effect could be attributed to the modulation of cytokine expression by the brain–gut axis, which would in-turn, downregulate the inflammatory response [55,56]. These results demonstrate that TNF-alpha is indeed implicated in all these diseases and that infliximab targets TNF-alpha downstream signaling.

**Figure 6:**
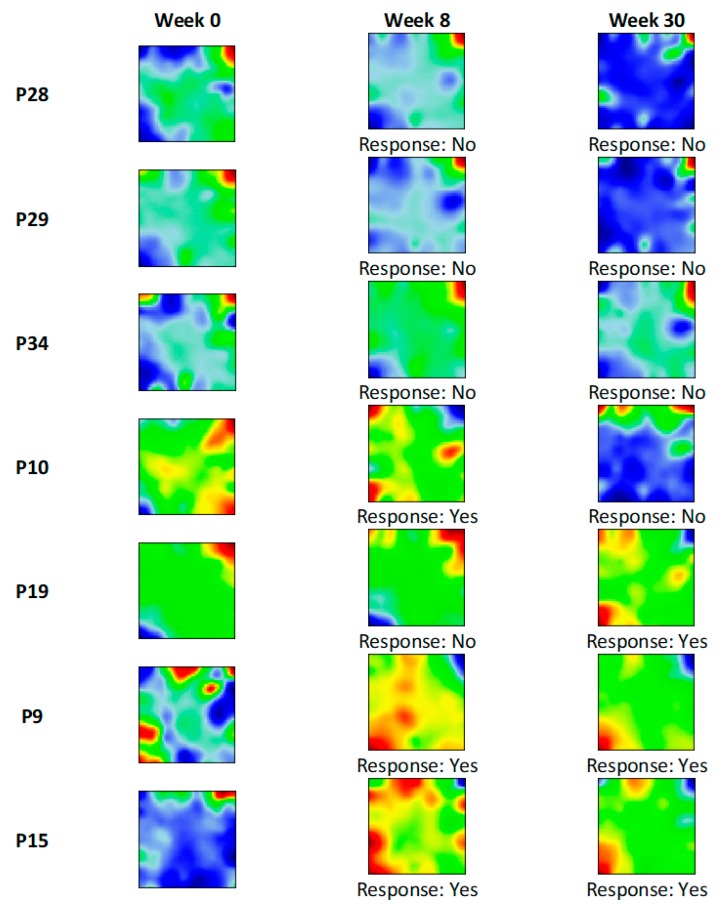
Infliximab response associated changes in SOM portraits in ulcerative colitis (UC) patients. Infliximab dataset (GSE23597) contained paired colonic biopsy microarray data obtained at week 0 (baseline) and week8, week 30, after treatment. This allowed for tracing how transcriptome landscapes change depending on infliximab response efficacy. Baseline and non-responder portraits contain distinctive red (overexpressed) spots in the top right and blue (underexpressed) spots in the bottom left. Responders were characterized by a reversed picture with overexpressed spots in the bottom left and underexpressed spots in the top right of the portrait.

Next, we generated transcriptome portraits from all samples using the ml-SOM approach (Supplementary Figures S2 and S3), which enabled us to visually examine the dynamics of drug response associated alterations of the transcriptome in paired colonic biopsy samples collected at week 0 (baseline), week 8, and week 30 after infliximab treatment. No changes in spot distribution were seen in SOM portraits of non-responders, while the spot patterns reversed in responders compared to baseline this way, indicating drug effect (Figure 6).

**Figure 7:**
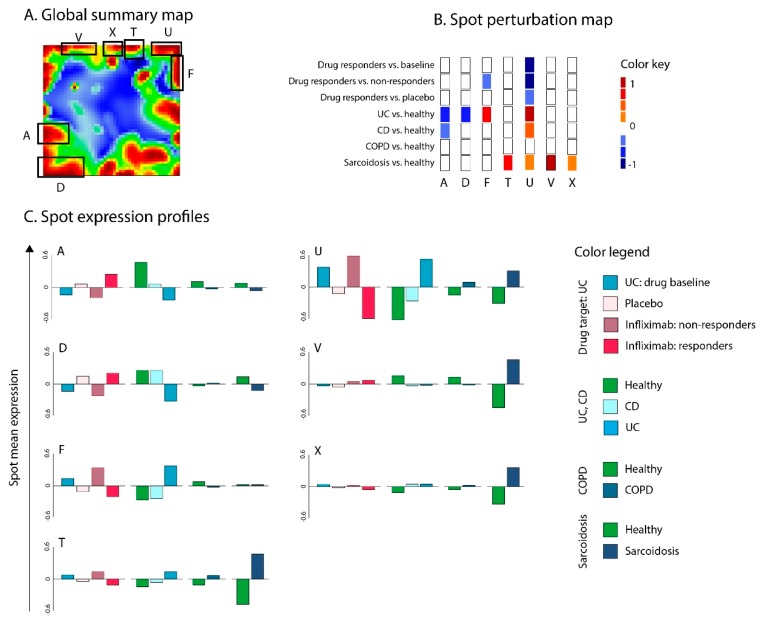
Evaluation of infliximab (T1) as a potential therapeutics for UC (D1), Crohn’s disease (CD) (D2), COPD (R1), and sarcoidosis (R2). (**A**) Global transcriptome summary map. Co-regulated gene clusters form spot-like patterns. (**B**) Drug- and disease-spot perturbation heatmap. (**C**) Spot expression profiles. The analysis results suggest that spot U, which was upregulated in UC, CD, and sarcoidosis, can be antagonistically targeted by infliximab. In addition, spot F, which was upregulated in UC, is associated with response to infliximab. Other disease-associated spots (A, T, V, X) did not overlap with infliximab-associated spots.

For an overview, all the spots were combined into a global summary map (Figure 7A), which can be interpreted as the transcriptome landscape of all samples from all layers. Overall, 25 spots were identified and labeled with letters A to Y. Based on the spot-calling strategy described in the Methods section, seven spots were differentially regulated in target versus reference groups (diseases versus healthy controls or infliximab action versus disease baseline). Functional annotation showed that these spots collect genes related to a wide range of biological functions (Table 1).

Next, we generated a spot perturbation heatmap of dysregulated spots to evaluate the “repositioning” potential of infliximab (Figure 7B). Spot perturbation heatmaps showed that spot U was significantly upregulated in UC compared to healthy controls, but was downregulated in the drug responder group of UC compared to non-responders or baseline groups. Therefore, it was considered a “drug target” spot (Figure 7B). This spot collects genes associated with the immune/inflammatory response and TNF-alpha/interferon signaling downstream activities in agreement with the results presented in the previous subsection (Table 1 and Figure 7C). In addition to UC, this spot showed marked upregulation in CD and sarcoidosis samples, which suggests that infliximab can also effectively target these diseases.

In addition, spot F, which was also upregulated in UC, was dysregulated in infliximab responder vs. non-responder groups. This spot was also associated with biological processes that amplify inflammation, such as cell and focal adhesion, collagen catabolic processes, activation of complement. However, this spot was not differentially dysregulated in responders vs. placebo or responders vs. baseline groups, which did not qualify it as a drug target spot. Spot F was upregulated in UC but not in other diseases. Hence, this infliximab can have a specific extended effect in UC. No dysregulated spots were observed for COPD, indicating that infliximab is presumably unsuited for the treatment of this disease, in contrast to UC, CD, and sarcoidosis. Notably, the expression of genes related to TNF receptor binding showed the smallest effect in COPD in support of this result (Figure 5).

In addition to drug target spots F and U, UC was also characterized by downregulation of spots A and D, and CD, by the downregulation of spot A. These spots collected intestine gene signatures, as well as gene sets related to digestion, transport, and metabolism of various nutrients (Table 1), demonstrating the loss of function of gastrointestinal tract epithelium [57]. Finally, evaluation of spot perturbation profiles in sarcoidosis showed upregulation of the other three spots (T, V, and X; Figure 7B); however, none of those spots were specifically linked to TNF-alpha mediated immune/inflammatory response. Hence, expression spot analysis revealed different pathogenic mechanisms related to the different diseases considered: Specifically, it filters them with regard to common responses to infliximab according to its action on TNF-pathway.

### 3.2. Evaluation of Brodalumab as a Potential Therapeutics for Psoriasis, Crohn’s Disease, and Systemic Juvenile Idiopathic Arthritis

**Figure 8:**
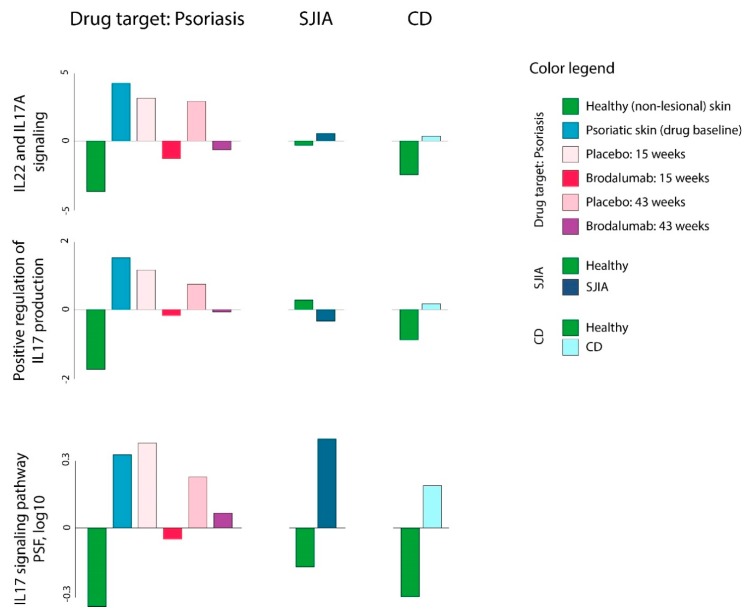
Gene set Z-score and PSF analysis of IL-17 associated gene sets in psoriasis (D1), systemic juvenile idiopathic arthritis (SJIA) (R1), CD (R2) and brodalumab (T1) datasets. IL-17 associated gene sets were chosen from gene set collection available in the oposSOM package [17].

In the next series of analyses, we aimed to evaluate the effects of brodalumab (T1) in psoriasis (D1), CD (D1), and SJIA (D2). Brodalumab is a human IgG2 monoclonal antibody against IL-17 receptor A (IL-17RA), which selectively inhibits IL-17 signaling [58]. This drug was shown to be capable of efficient blocking exacerbated inflammation in keratinocytes and leukocytes and cause improvement in clinical and histological characteristics in psoriatic patients [33,59]. As brodalumab targets IL-17 associated signal transduction, we first evaluated GSZ-scores and PSF values for corresponding gene sets associated with IL-17 and IL-22 signaling. Psoriasis and, to a lesser extent CD and SJIA, were characterized by overexpression of IL-17-associated gene sets, and, on the other hand, their downregulation in response to brodalumab compared with untreated psoriasis and placebo groups (Figure 8, Supplementary Figure S4).

**Figure 9:**
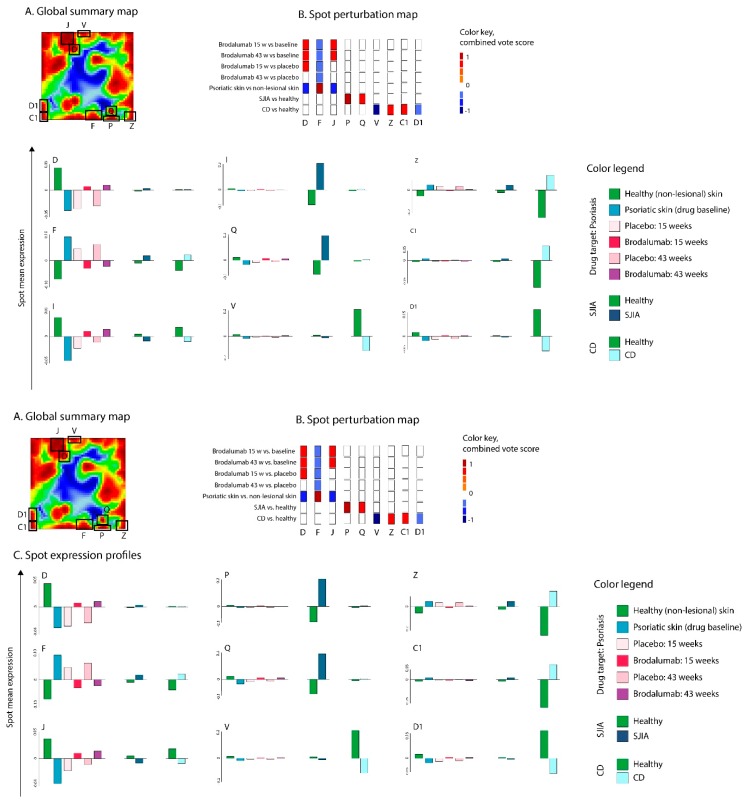
Evaluation of brodalumab (T1) as a potential therapeutics for psoriasis (D1), CD (R1), and SJIA (R2). (**A**) Global transcriptome summary map. Co-regulated gene clusters formed spot-like patterns. (**B**) Drug– and disease–spot perturbation heatmap. (**C**) Spot expression profiles. The analysis results suggest that brodalumab antagonized the deregulation in psoriasis-associated spots D, F, and J. Other diseases (CD and SJIA) associated spots (P, Q, V, Z, C1, D1) did not overlap with brodalumab-associated spots.

SOM portrayal (Supplementary Figures S5 and S6) and spot detection segmented global transcriptome landscape of psoriasis, CD, and SJIA into 35 spots (Figure 9A), from which only nine (Figure 9C) were dysregulated in target groups (diseases, or drug) compared to the corresponding reference group (baseline, placebo or healthy control). The functional annotation of these spots is presented in Table 2.

Spot perturbation heatmap (Figure 9B) analysis identified spot (F) upregulation in psoriasis lesional skin compared to normal skin and which was downregulated in the brodalumab group compared to disease baseline and placebo. Spot F was associated with keratinization, keratinocyte differentiation, innate immune response, and biosynthesis of ceramide and sphingolipids metabolic processes. Meanwhile, spots D and J were upregulated in brodalumab group compared to baseline; however, no differences were observed in brodalumab vs. placebo groups. These spots were characterized by gene sets related to signal transduction, ion channel activity, actin-binding, and calcium signaling. Overall the results indicate that brodalumab efficiently targets IL-17 related pathways. However, its action is much broader than just inhibition of IL-17 associated immune response in agreement with [33,59].

Transcriptome landscape for SJIA in peripheral blood was characterized by the upregulation of spots P and Q. These spots collect functional terms associated with an acute response, inflammation, neutrophil degranulation, and erythrocyte differentiation, which is in line with previous observations of prominent upregulation of erythropoiesis related gene-expression [60] and innate immune response [61,62].

CD, in turn, was characterized by downregulation of spots V and D1 in colon tissue samples, that collect genes related to the ciliary membrane, pancreatic beta-cell differentiation, digestion, transport, and lipid metabolic processes. On the other side, spot Z, upregulated in CD, collected gene sets related to activated inflammatory and immune response, chemotaxis, neutrophil degranulation, and response to interferon-gamma. Interestingly, genes located in spot Z largely overlapped with infliximab target genes, as was described in the previous section. In addition, upregulated spot C1 in CD collected genes linked to bile acid and bile salt transport, lipid metabolism, and oxidation−reduction processes.

Thus, in contrast to the favorable action of brodalumab in psoriasis, our analysis demonstrated that this drug will not interfere with pathological processes in SJIA and CD since no dysregulation was observed in drug target spots F, D, and J in both SJIA and CD.

### 3.3. Result Validation with CMap

We used the Connectivity Map (CMap) [9] to validate the results of the ml-SOM analyses. The CMap platform provides an interface to determine the association of drugs with a given list of dysregulated genes via enrichment analysis of gene expression with drug-driven gene profiles [9]. Neither infliximab nor brodalumab was included in the drug collection in CMap. Therefore, we performed a two-step analysis to validate our findings against the CMap algorithm. First, we performed differential gene expression analysis to identify the top 150 (the maximum number handled by CMap) downregulated genes by infliximab and brodalumab, and then queried these lists against the reference perturbagen signatures in CMap. Then, we queried CMap with top 150 genes from the “drug” spots identified by ml-SOM (spot U for infliximab, and spots D and F for brodalumab). Finally, we performed the same analyses with the top 150 upregulated gene lists in diseases. These queries produced scores for 171 compound classes, reflecting the similarity of supplied gene lists with reference gene set lists in CMap. Next, we performed pairwise correlations of CMap scores. This provided us with an indirect measure of the association between drug-, spot- and disease-associated gene lists, according to the CMap algorithm. The results showed that the CMap compound scores for infliximab were correlated with those of UC, CD, and sarcoidosis, but not with COPD (Supplementary Figure S7). Moreover, correlation scores increased for sarcoidosis but not for COPD, when instead of differentially expressed gene lists, we considered the drug spot-genes for infliximab. These results indicate that ml-SOM better selects specific drug effects compared with standard two-group differential gene expression analysis. Similarly, brodalumab profiles showed a marked correlation with psoriasis, but not with SJIA and CD. Accounting for drug spot-genes did not increase the correlation with SJIA and CD (Supplementary Figure S8). These results confirm the results of our ml-SOM algorithm against that of CMap.

## 4. Discussion

Drug repositioning opens new possibilities for the fast delivery of new treatment options by reducing the time and resources spent on drug development and testing [1,2]. Systematic analysis of concerted transcriptome changes in response to disease and drug action provides a useful concept in this field [63,64,65]. However, it is essential to consider gene expression changes on the whole transcriptome level and not only identify drug–target processes or genes to improve the predictive power of the method but also to understand how off-target genes may interfere with disease development, drug action or be related to the risk of adverse effects.

In this study, we presented a novel approach to drug repositioning based on the evaluation of full transcriptome landscapes of drug action on diseased states utilizing the multilayer self-organizing maps (ml-SOM) machine learning technique. ml-SOM segments layers of drug action- and disease-associated transcriptome landscapes into clusters co-regulated and thus functionally related, which are identified as spots with similar localization across different layers. This allows for direct comparison of their expression changes across the layers and identification of “drug target” spots in disease layers, as well as of similarities of the functional context of drug target and non-target spots. We applied the ml-SOM approach to evaluate the possibility of repositioning biologics infliximab (in COPD and sarcoidosis) and brodalumab (in CD and SJIA).

Infliximab has been proven to be efficient in UC and CD by blocking TNF-alpha activity [47,48,49]. Our results confirmed the mechanism of infliximab action, including induction of apoptosis in T cells, inhibition of cytokine and chemokine expression, as well as cell–cell signaling and immune cell trafficking [27], and its efficacy in these diseases. Our ml-SOM method predicted the repositioning potential of infliximab in CD [49] based on its effect in UC, which can be considered as a retrospective validation of our methodology. On the other hand, systemic inflammation and TNF-alpha response have also been implicated in COPD and sarcoidosis. It has been shown that a number of single nucleotide polymorphisms in the TNF-alpha promoter were associated with an increased risk of these diseases [23,24,25]. Furthermore, elevated levels of affected cytokines were observed in blood, bronchial biopsies, as well as bronchoalveolar lavage of COPD patients [26]. Our results on TNF-alpha related gene set enrichment analysis also confirmed these observations (Figure 4). However, with our ml-SOM approach, we could show that infliximab successfully targets these gene sets in UC, CD, and sarcoidosis, but not in COPD. It seems that alternative mechanisms not targeted by infliximab support exacerbation of inflammation and COPD symptom development, confirming little to no effect of infliximab on the improvement of COPD course in a number of trials [66,67]. Our results, on the other side, suggest that there is a potential benefit of using infliximab in sarcoidosis since it can target immune system-related gene modules upregulated in the disease course. These findings are in line with previous publications suggesting infliximab as an off-label indication for sarcoidosis [68,69,70,71].

Brodalumab is another biologics (anti-IL-17 receptor A antibody) drug that was recently approved by the FDA for the treatment of severe-to-moderate psoriasis and psoriatic arthritis [30]. Since IL-17 signaling is also implicated in the pathophysiology of Crohn’s disease (CD) [31] and systemic juvenile idiopathic arthritis (SJIA) [32], we evaluated brodalumab repositioning potential for these diseases. The results of ml-SOM confirmed the action of brodalumab in psoriasis that includes inhibition of keratinization in epidermal cells and leveraging immune response at the site of psoriatic lesions [33,59]. In contrast to psoriasis, we did not observe the dysregulation of brodalumab target genes in SJIA and CD. The functional annotation of gene clusters (spots) associated with SJIA showed upregulation of gene sets related to acute response, inflammation, neutrophil degranulation, and erythrocyte differentiation. Moreover, recent reports indicate that SJIA can be considered as an autoinflammatory rather than an autoimmune disease that is driven by T-cell dysregulation [72,73]. While results on T-cell activation during SJIA are conflicting [74,75], it has been shown that SJIA patients respond well to IL-1 antagonists [76], which supports the autoinflammatory nature of the disease. Though there are no studies of the efficacy of the drug in SJIA, it should be noted that no effect for brodalumab was recorded for the drug in rheumatoid arthritis [77]. Thus, our results suggested little to no efficacy of brodalumab for SJIA treatment. Our results also showed that brodalumab will not be efficient in CD either. Indeed, recent trials have shown that brodalumab worsens the clinical course of CD [78]. Moreover, in psoriasis trials, some patients have developed CD during the treatment course, and according to the FDA, CD is included in contraindications for this drug [79].

Using CMap as a validation approach, we obtained concordant results. CMap, as well as NFFinder, offers “signature” based analysis, i.e., comparison of user-supplied list against pre-populated “reference” lists of gene perturbations. CMap has limits on the number of input genes (max 150 genes). Both CMap and NFF assess the “similarity” of gene expression profiles to a “predefined” list of compounds. ml-SOM allows for direct analysis of selected drug/biologics in selected diseases and provides lists of candidate genes. CMap uses the Kolmogorov-Smirnov enrichment statistic score [39] for assessment of profile similarity, while NFFinder uses the page rank algorithm [80]. ml-SOM uses self-organizing maps to cluster genes to modules on a transcriptome map based on expression profiles across samples. This modularization of the expression landscape into groups of co-regulated (and thus functionally related) genes makes ml-SOM a powerful selection tool for candidate gene search in drug repositioning tasks. Note that ml-SOM extracts the list of drug-responsible genes, which is required as input for CMap analysis for drug effects. Hence, ml-SOM can also be effectively combined with CMap for the evaluation of drug-repositioning.

The latter results indicate that our approach could also be used to identify possible drug adverse effects on target and repositioned diseases. Indeed, the ml-SOM analysis showed that the expression of gene modules targeted by brodalumab (spots D and F) were not dysregulated in CD (Figure 9). This could mean that brodalumab would further downregulate these modules in the disease. Spots D and F contained target processes associated with IL17, and their downregulation, along with the elevated inflammatory response (spot Z), could be a reason for exacerbation of CD symptoms. Indeed, IL17 is crucial for the recruitment of rTh 17 cells that can suppress inflammation in the gut [81]. In addition, IL17 suppression could elevate inflammation in the gut by disrupting epithelial integrity and favoring increased leakage of microorganisms [82]. In a more generalized way, the “drug target spots” on transcriptome portraits could be considered to cause adverse effects if they are differentially regulated upon treatment but not in disease. Unfortunately, there is limited data on the role of genes in adverse effect formation beyond the drug-metabolizing system, but recently this is becoming a focus area of research [83,84], and the growing annotation data could further be integrated into the ml-SOM pipeline. Another important advantage of ml-SOM is the possibility of “personalizing” the assessment of drug repositioning. In the case of infliximab repositioning, spot perturbation heatmaps show that downregulation of spot F along with the upregulation of spot U was associated with the response to the drug (Figure 7B,C). Thus, we can speculate here, that “screening for drug target spots” in sample-specific SOM molecular portraits could be used to predict the drug responses on the levels of an individual sample.

All in all, the ml-SOM approach proved to be efficient in silico methodology for drug repositioning. Its main advantage is the ability of a holistic analysis of transcriptome landscapes in different conditions and their direct comparison. From a performance point of view, the number of data layers that can be handled by ml-SOM is limited only by computational resources. In addition, datasets with small numbers of samples can be used with minimum sampling bias, since they are combined in a larger final dataset. At the same time, ml-SOM is robust towards batch effects since training considers the profile of gene expression across layers, while differential spot calling is applied on each layer separately. Compared to other methods, the ml-SOM approach is able to not only identify disease-drug pairs but also predict drug side effects, as well as provide functional information on the molecular processes that drive drug–disease and drug–side effect associations. Finally, this approach opens the possibility of performing a “personalized” assessment of drug repositioning based on individual, sample-specific SOM molecular portraits.

## 5. Conclusions

With the increasing availability of various drug action and disease-associated whole transcriptome data, it has become an attractive source for drug repositioning studies. Our ml-SOM approach not only extends the limited battery of available transcriptome-guided repositioning tools but also offers more extended data analysis, interpretation, and visualization capabilities. The strength of our method is its ability to evaluate changes in the transcriptome in response to disease and drugs across multiple transcriptome datasets, identification of drug effect linked genes clusters and functional processes, as well as drug response and adverse effects predictions. We believe that our approach will be particularly useful for repositioning of biologics that are currently replacing small molecules as the main options for therapy in many complex diseases.

## Figures and Tables

**Table 1:** Overrepresentation analysis of dysregulated spots (top 3 most significant gene sets).

Spot	Gene Set	*p*	FDR	GS ^1^ Type	GS Reference
A	protein binding	3 × 10^−11^	2 × 10^−9^	GO MF ^2^	[38]
	bicellular tight junction assembly	3 × 10^−10^	1 × 10^−8^	GO BP ^3^	[38]
	positive regulation of epithelial cell proliferation	1 × 10^−7^	7 × 10^−6^	GO BP	[38]
D	small intestine	2 × 10^−32^	4 × 10^−30^	RS ^4^	[40]
	duodenum	7 × 10^−31^	1 × 10^−28^	RS	[40]
	lipid metabolic process	1 × 10^−25^	2 × 10^−23^	GO BP	[38]
F	extracellular matrix organization	1 × 10^−37^	1 × 10^−35^	GO BP	[38]
	cell adhesion	3 × 10^−32^	3 × 10^−30^	GO BP	[38]
	angiogenesis	8 × 10^−22^	5 × 10^−20^	GO BP	[38]
T	immune related cell surface molecules	1 × 10^−13^	1 × 10^−10^	RS	[41]
	germinal center B-cells	1 × 10^−11^	1 × 10^−8^	RS	[18]
	immune system process	6 × 10^−7^	0.0001	GO BP	[38]
U	immune system	1 × 10^−98^	2 × 10^−95^	RS	[36]
	immune system process	9 × 10^−86^	1 × 10^−82^	GO BP	[38]
	immune response	3 × 10^−79^	3 × 10^−76^	GO BP	[38]
	TNFA signaling via NFKB	2 × 10^−31^	4 × 10^−29^	HALLMARK ^5^	[36]
V	protein phosphatases	2 × 10^−17^	2 × 10^−15^	RS	[41]
	nucleic acid binding	3 × 10^−15^	2 × 10^−13^	GO BP	[38]
	metabolism	2 × 10^−12^	1 × 10^−10^	RS	[41]
X	cytosceleton	1 × 10^−8^	1 × 10^−6^	RS	[41]
	protein binding	5 × 10^−7^	3 × 10^−5^	GO BP	[38]

^1^ GS—Gene set; ^2^ GO MF—Gene Ontology molecular function; ^3^ GO BP—Gene Ontology biological process; ^4^ RS—Reference signatures, ^5^ HALLMARK—Cancer hallmarks.

**Table 2:** Overrepresentation analysis of dysregulated spots (top 3 most significant gene sets).

Spot	Gene Set	*p*	FDR	GS ^1^ Type	GS Reference
	nervous system	2 × 10^−12^	3 × 10^−10^	RS ^2^	[36]
D	ion transmembrane transport	8 × 10^−6^	0.0008	GO BP ^3^	[38]
	Reactome transmembrane transport of small molecules	1 × 10^−5^	0.001	GSEA ^4^	[39]
	cornification	1 × 10^−18^	5 × 10^−16^	GO BP	[38]
F	keratinization	7 × 10^−15^	2 × 10^−12^	GO BP	[38]
	keratinocyte differentiation	1 × 10^−13^	3 × 10^−11^	GO BP	[38]
	nervous system	1 × 10^−12^	0.99	RS	[36]
J	calcium-dependent cell-cell adhesion via plasma membrane cell adhesion molecules	5 × 10^−9^	1.00	GO BP	[38]
	homophilic cell adhesion via plasma membrane adhesion molecules	1 × 10^−7^	1.00	GO BP	[38]
	neutrophil degranulation	1 × 10^−17^	1 × 10^−14^	GO BP	[38]
P	primary lymphoid organs	2 × 10^−12^	1 × 10^−9^	RS	[36]
	defense response to bacterium	4 × 10^−11^	2 × 10^−8^	GO BP	[38]
	endosome to melanosome transport	0.0004	0.04	GO BP	[38]
Q	neutrophil degranulation	0.0004	0.04	GO BP	[38]
	negative regulation of macrophage derived foam cell differentiation	0.0006	0.06	GO BP	[38]
	endocrine pancreas development	1 × 10^−6^	0.0002	GO BP	[38]
V	type B pancreatic cell development	2 × 10^−6^	0.0002	GO BP	[38]
	Reactome regulation of beta cell development	3 × 10^−6^	0.0003	GSEA	[39]
	inflammatory response	3 × 10^−41^	5 × 10^−38^	GO BP	[38]
Z	immune system process	2 × 10^−26^	1 × 10^−23^	GO BP	[38]
	immune response	2 × 10^−26^	1 × 10^−23^	GO BP	[38]
	bile acid and bile salt transport	1 × 10^−8^	9 × 10^−6^	GO BP	[38]
C1	Reactome biological oxidations	2 × 10^−7^	0.0001	GSEA	[39]
	KEGG retinol metabolism	1 × 10^−6^	0.00046	GSEA	[39]
	KEGG drug metabolism cytochrome p450	8 × 10^−9^	8 × 10^−7^	GSEA	[39]
D1	cellular response to zinc ion	1 × 10^−8^	9 × 10^−7^	GO BP	[38]
	negative regulation of growth	1 × 10^−8^	9 × 10^−7^	GO BP	[38]

^1^ GS—Gene set; ^2^ RS—Reference signatures; ^3^ GO BP—Gene ontology biological process; ^4^ GSEA—Gene set enrichment analysis gene sets.

## Supplementary Materials

The following are available online at https://www.mdpi.com/1999-4923/11/12/677/s1: Figure S1: Heatmap of activation profiles of selected KEGG signaling pathways in ulcerative colitis (D1), Crohn’s disease (D2), COPD (R1), and sarcoidosis (R2) as well as infliximab (T1) datasets. Figure S2: Individual SOM portraits in an infliximab (Study 1) repositioning study (see Materials and Methods, Experiment Design and Data Sets subsection). Figure S3: Infliximab repositioning study: mean group and representative individual SOM portraits. Figure S4. Heatmap of activation profiles of selected KEGG signaling pathways in psoriasis (D1), SJIA (R1), CD (R2), and brodalumab (T1) datasets. Figure S5: Individual SOM portraits in a brodalumab (Study 2) repositioning study (see Materials and Methods, Experiment Design and Data Sets subsection). Figure S6: Brodalumab repositioning study: mean group and representative individual SOM portraits. Figure S7. Comparison of perturbagen scores profiles for infliximab and UC, CD, COPD, and sarcoidosis. Figure S8. Comparison of perturbagen scores profiles for brodalumab and psoriasis, SJIA, CD.
