# Supplementary material for: Transcriptome-Guided Drug Repositioning
Source: Pharmaceutics. 2019 Dec 12;11(12):677. doi: 10.3390/pharmaceutics11120677 (PMC6969900; doi:10.3390/pharmaceutics11120677)
Supplement: Supplementary file 1 [file pharmaceutics-11-00677-s001.pdf]

pharmaceutics

Supplementary Materials: Transcriptome-Guided Drug Repositioning

Arsen Arakelyan, Lilit Nersisyan, Maria Nikoghosyan, Siras Hakobyan, Arman Simonyan, Lydia Hopp, Henry Loeffler-Wirth and Hans Binder

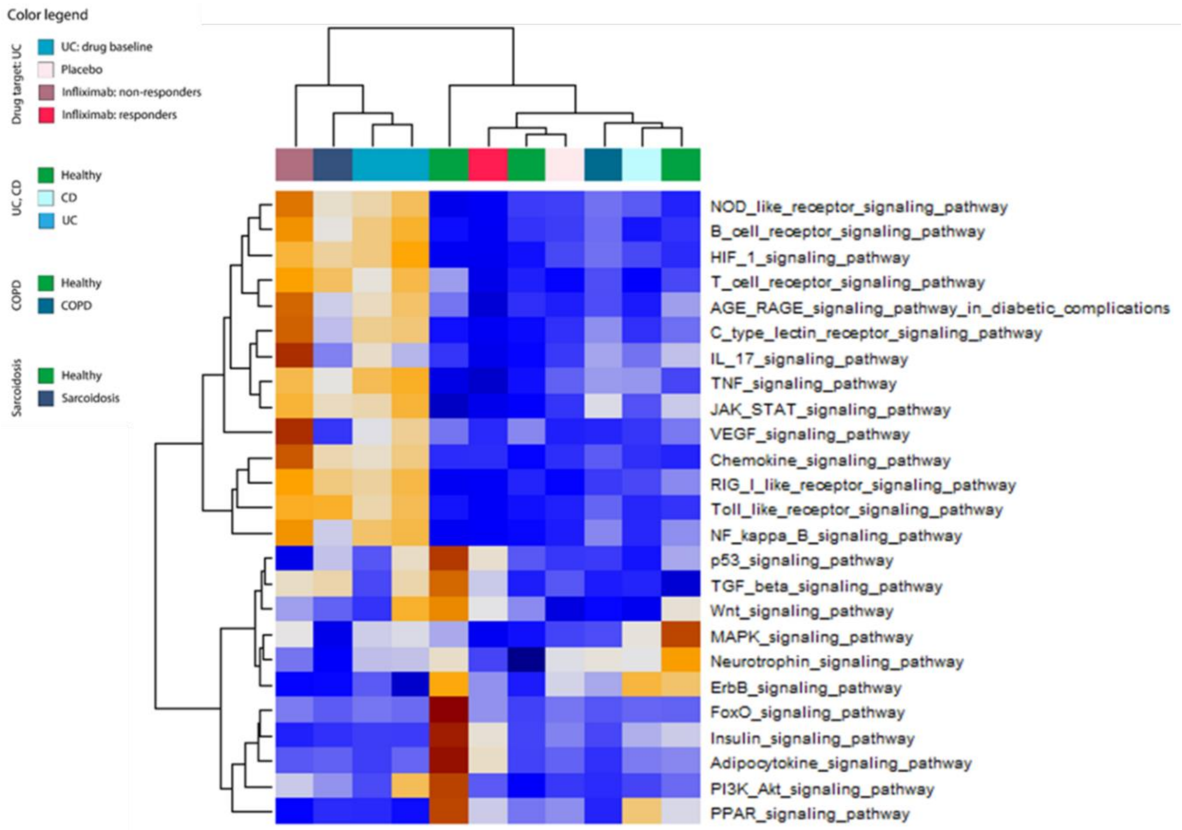

Figure S1. Heatmap of activation profiles of selected KEGG signaling pathways in ulcerative colitis (D1), Crohn's disease (D2), COPD (R1) and sarcoidosis (R2) as well as infliximab (T1) datasets. The activity of pathways was estimated using the pathway signal flow (PSF) method which takes into account the expression level of the genes which form the nodes in the pathway graph, and their mutual interactions, depicted in the pathway topology. The results show the increased activities in immune response and inflammation-related pathways in diseases and non-responders, while responder related profiles are clustered with healthy groups indicating the effect of the drug on TNF-alpha interlinked pathways.

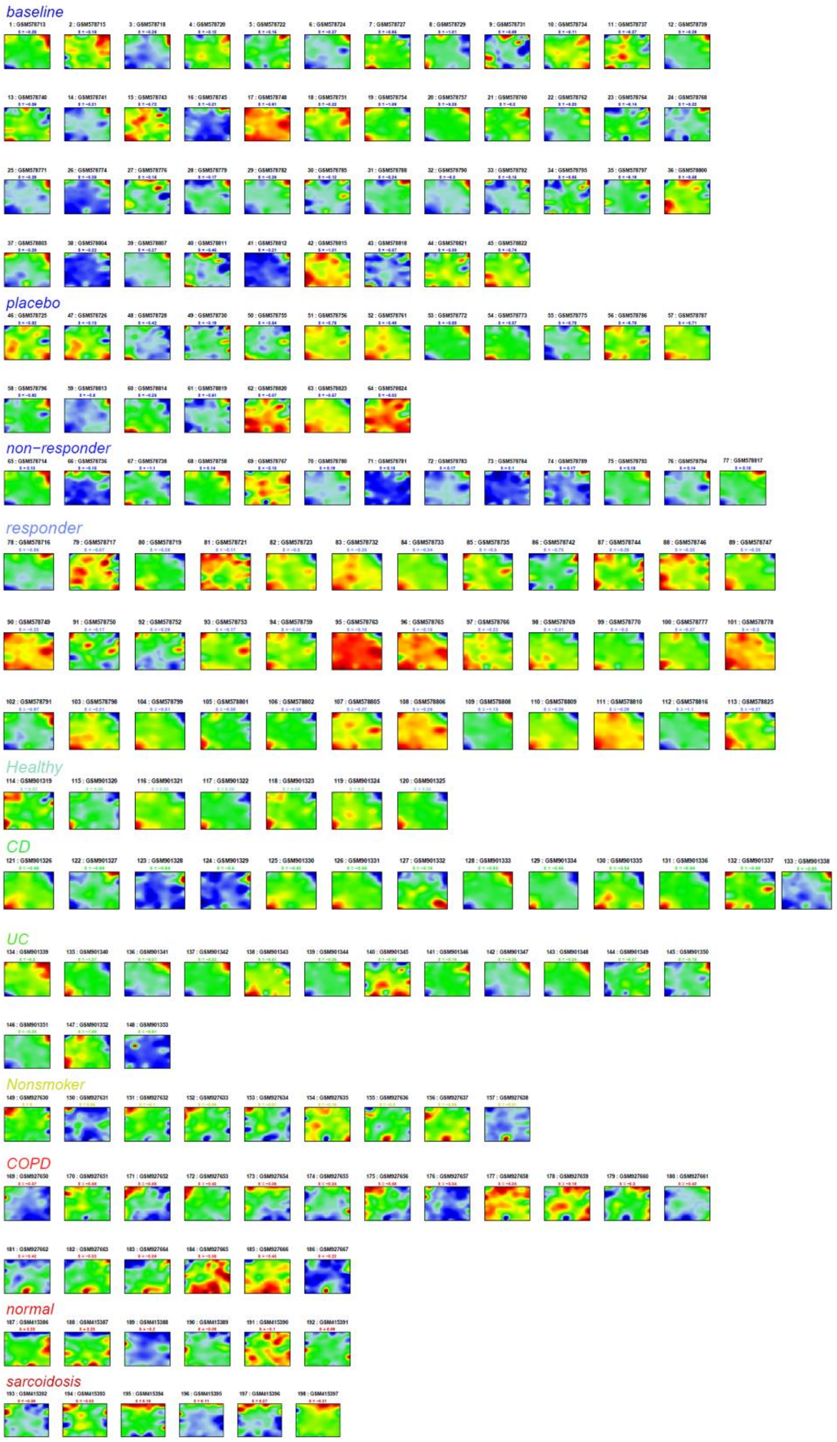

Figure S2. Individual SOM portraits in Infliximab (Study 1) repositioning study (see Materials and Methods, Experiment design and data sets subsection). Baseline—treatment baseline (patients not receiving infliximab), placebo—patients receiving placebo, non-responders—patients receiving infliximab, but not responding, responder—patients receiving infliximab with the positive response to treatment. Healthy—healthy controls, UC—patients with ulcerative colitis, CD—patients with Crohn's disease, Nonsmoker—healthy controls for COPD study, COPD—patients with COPD, normal – healthy controls for sarcoidosis patients, sarcoidosis—patients with sarcoidosis.

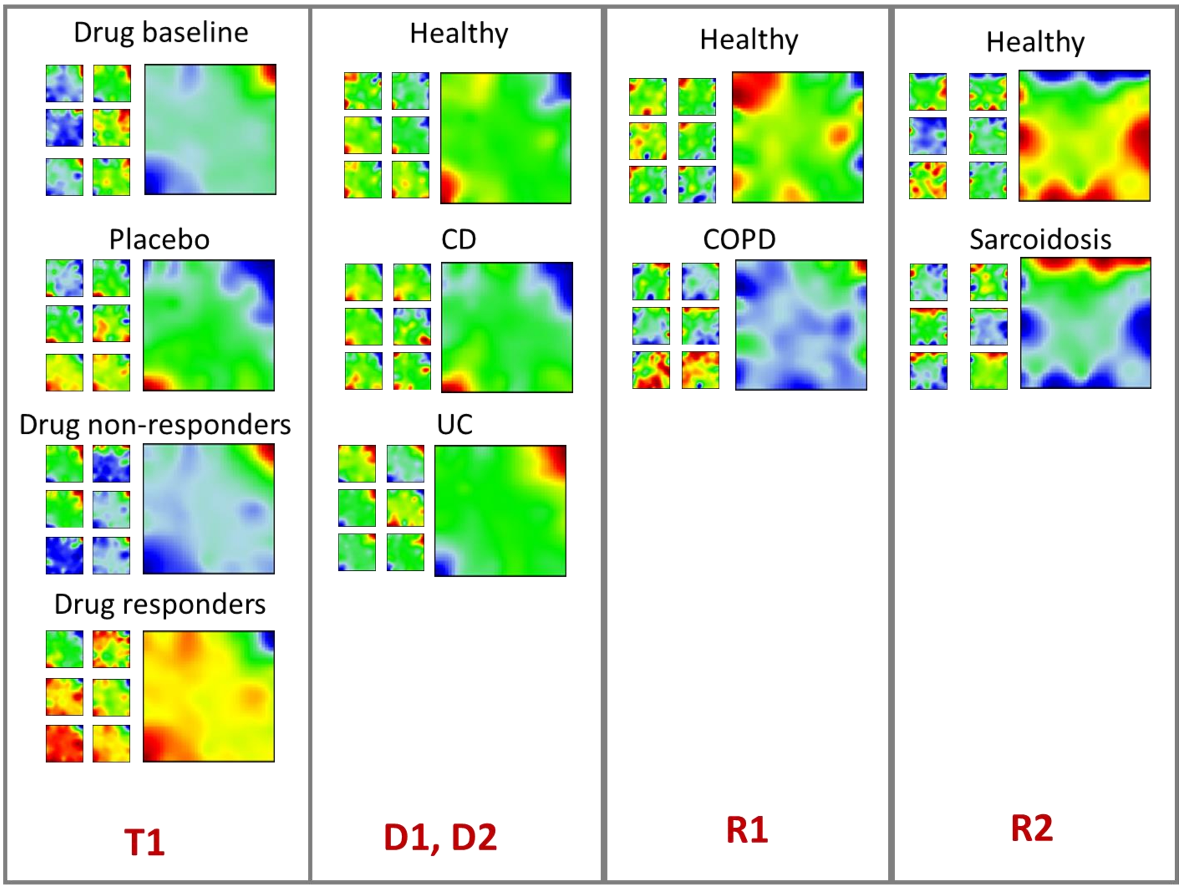

Figure S3. Infliximab repositioning study: mean group and representative individual SOM portraits. T1—treatment (Infliximab) dataset, D1 and D2—target disease datasets for T1, R1 and R2—repositioning disease data sets.

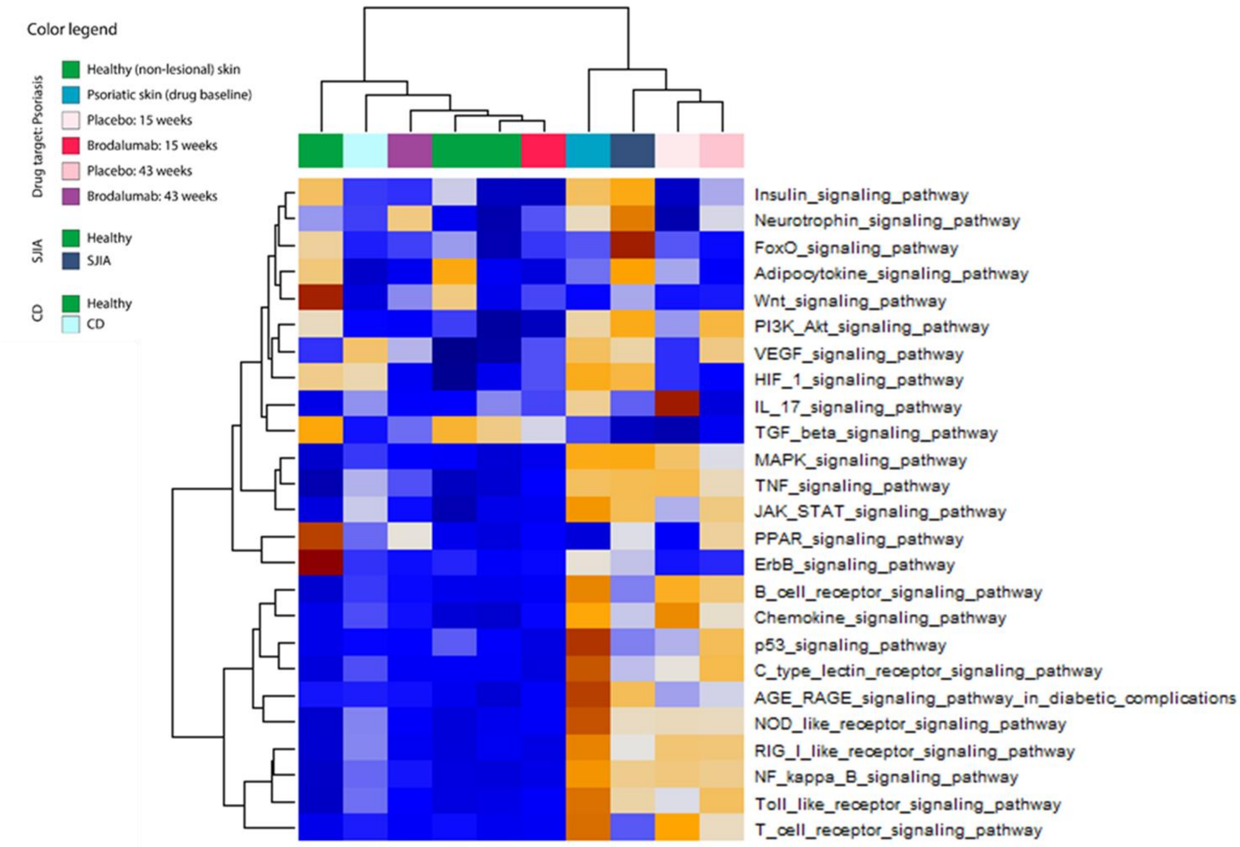

Figure S4. Heatmap of activation profiles of selected KEGG signaling pathways in psoriasis (D1), SJIA (R1), CD (R2) and brodalumab (T1) datasets. The activity of the pathways was estimated using the pathway signal flow (PSF) method which takes into account the expression level of the genes which form the nodes in the pathway graph, and their mutual interactions, depicted in pathway topology. The results show increased activities of IL-17 related KEGG pathways in diseases and in placebo groups, while brodalumab responders cluster with healthy groups.

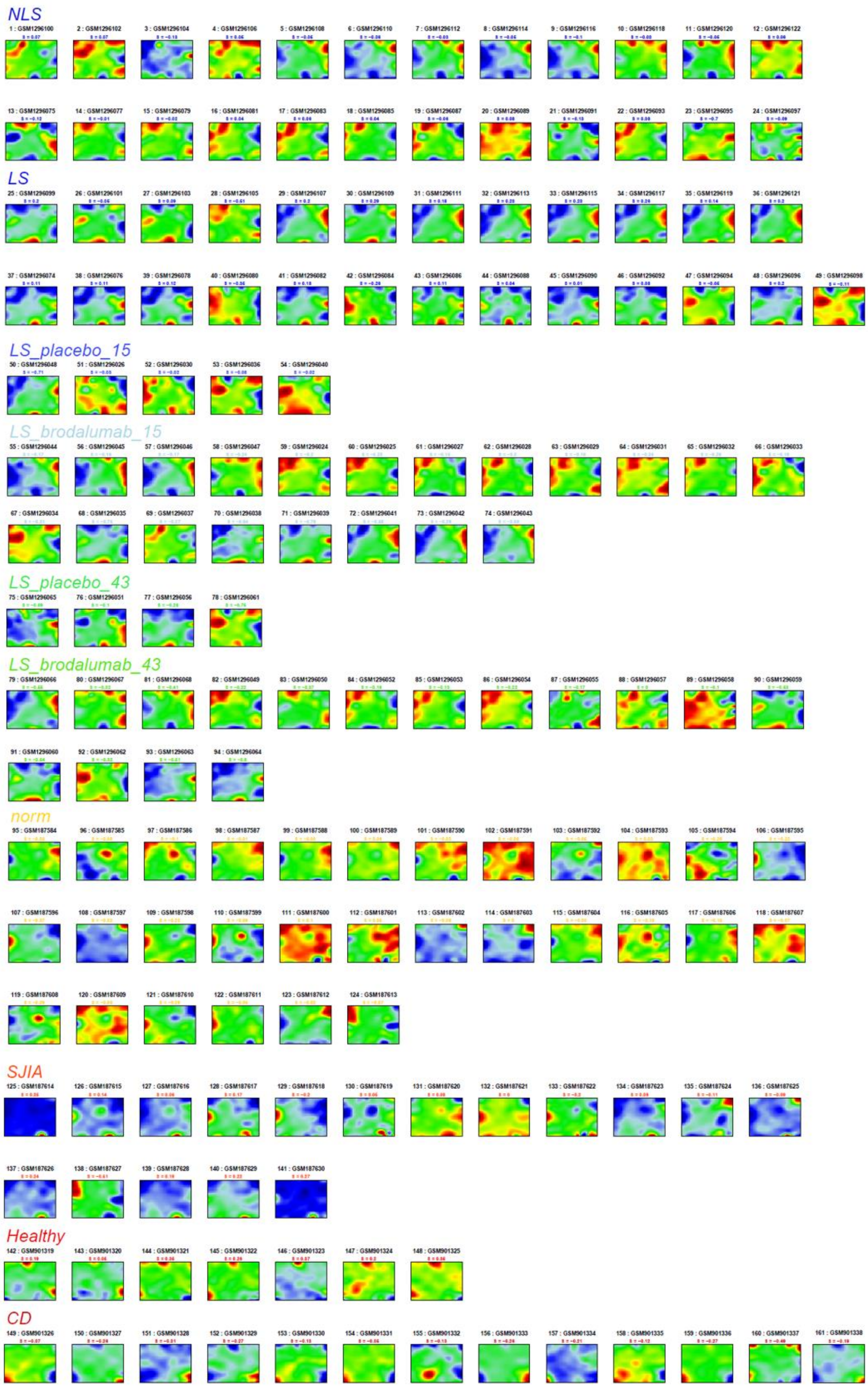

Figure S5. Individual SOM portraits in Brodalumab (Study 2) repositioning study (see Materials and Methods, Experiment design and data sets subsection). NLS—non-lesional skin biopsy, LS—psoriasis skin biopsy, LS_placebo_15—psoriasis patients receiving placebo on week 15, LS_brodalumab_15—psoriasis patients receiving brodalumab on week 15, LS_pacebo_43—psoriasis patients receiving placebo on week 43, LS_ brodalumab_43—psoriasis patients receiving placebo on week 43, norm—healthy controls for SJIA study, SJIA—systemic juvenile arthritis patients, Healthy—healthy controls for CD study, CD—patients with Crohn’s disease.

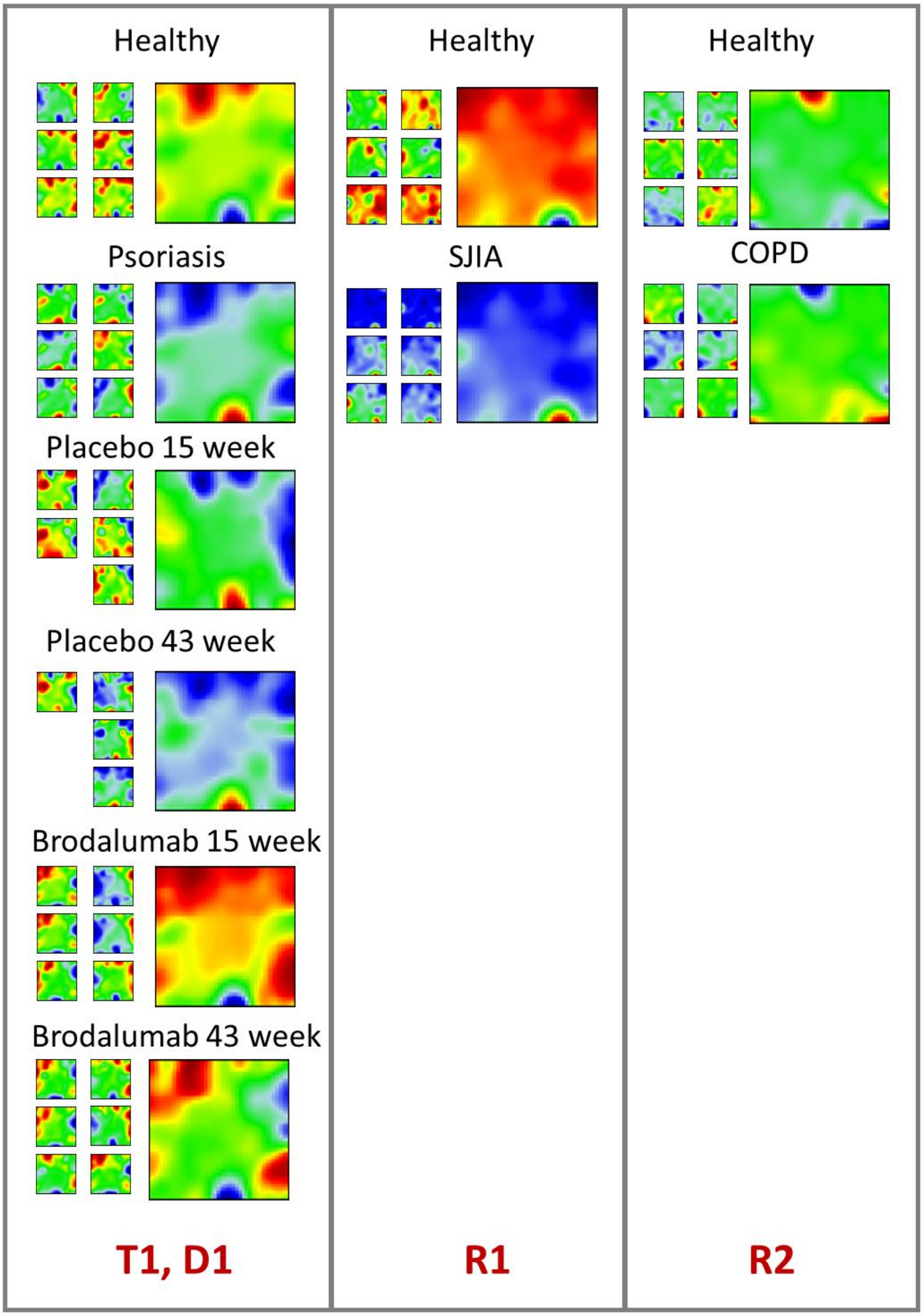

Figure S6. Brodalumab repositioning study: mean group and representative individual SOM portraits. T1—treatment (Brodalumab) dataset, D1—target disease dataset for T1, R1 and R2—repositioning disease data sets.

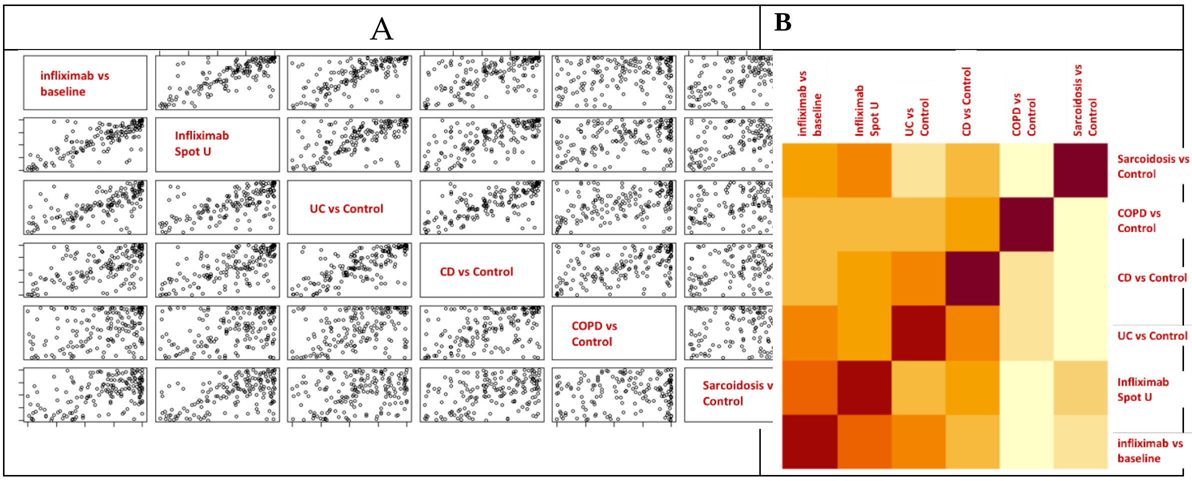

Figure S7. Comparison of perturbagen scores profiles for infliximab and UC, CD, COPD, and sarcoidosis. A) Scatterplot Matrices of CMap perturbagen score profiles for top150 differentially expressed genes for drug and diseases as well as infliximab spot U. B) Correlation heatmap for CMap perturbagen score profiles. The results of show correlation between infliximab, infliximab spot U, UC, CD and sarcoidosis. Moreover, correlation value for spot U is higher for sarcoidosis than that of top 150 differentially expressed genes.

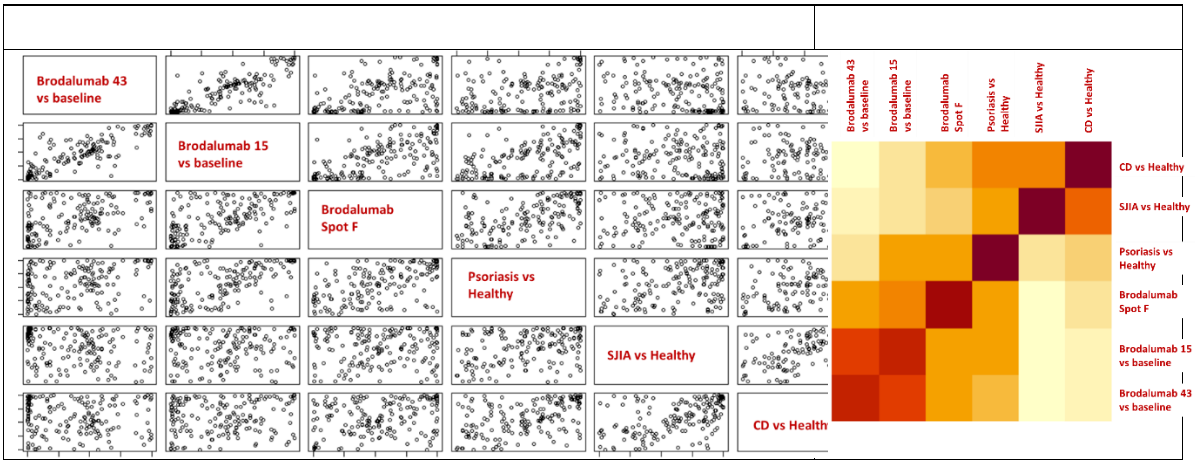

Figure S8. Comparison of perturbagen scores profiles for brodalumab and psoriasis, SJIA, CD. A) Scatterplot Matrices of CMap perturbagen score profiles for top150 differentially expressed genes for drug and diseases as well as brodalumab spot F. B) Correlation matrix for CMap perturbagen score profiles. The results of analysis show correlation between brodalumab and psoriasis, while no correlation was observed for SJIA and CD whatsoever.
